# Quantifying the Dosimetric Impact of Proton Range Uncertainties on RBE-Weighted Dose Distributions in Intensity-Modulated Proton Therapy for Bilateral Head and Neck Cancer

**DOI:** 10.3390/curroncol31070272

**Published:** 2024-06-27

**Authors:** Suresh Rana, Noufal Manthala Padannayil, Linh Tran, Anatoly B. Rosenfeld, Hina Saeed, Michael Kasper

**Affiliations:** 1 Department of Radiation Oncology, Lynn Cancer Institute, Boca Raton Regional Hospital, Baptist Health South Florida, Boca Raton, FL 33486, USA; 2 Department of Radiation Oncology, Florida International University, Miami, FL 33199, USA; 3 Centre for Medical Radiation Physics (CMRP), University of Wollongong, Wollongong, NSW 2522, Australia

**Keywords:** IMPT, head and neck cancer, variable RBE, RBE-weighted dose, proton therapy

## Abstract

Background: In current clinical practice, intensity-modulated proton therapy (IMPT) head and neck cancer (HNC) plans are generated using a constant relative biological effectiveness (cRBE) of 1.1. The primary goal of this study was to explore the dosimetric impact of proton range uncertainties on RBE-weighted dose (RWD) distributions using a variable RBE (vRBE) model in the context of bilateral HNC IMPT plans. Methods: The current study included the computed tomography (CT) datasets of ten bilateral HNC patients who had undergone photon therapy. Each patient’s plan was generated using three IMPT beams to deliver doses to the CTV_High and CTV_Low for doses of 70 Gy(RBE) and 54 Gy(RBE), respectively, in 35 fractions through a simultaneous integrated boost (SIB) technique. Each nominal plan calculated with a cRBE of 1.1 was subjected to the range uncertainties of ±3%. The McNamara vRBE model was used for RWD calculations. For each patient, the differences in dosimetric metrices between the RWD and nominal dose distributions were compared. Results: The constrictor muscles, oral cavity, parotids, larynx, thyroid, and esophagus showed average differences in mean dose (D_mean_) values up to 6.91 Gy(RBE), indicating the impact of proton range uncertainties on RWD distributions. Similarly, the brachial plexus, brain, brainstem, spinal cord, and mandible showed varying degrees of the average differences in maximum dose (D_max_) values (2.78–10.75 Gy(RBE)). The D_mean_ and D_max_ to the CTV from RWD distributions were within ±2% of the dosimetric results in nominal plans. Conclusion: The consistent trend of higher mean and maximum doses to the OARs with the McNamara vRBE model compared to cRBE model highlighted the need for consideration of proton range uncertainties while evaluating OAR doses in bilateral HNC IMPT plans.

## 1. Introduction

The recognition of intensity-modulated proton therapy (IMPT) as an advanced modality in radiation therapy for the management of head and neck (HN) cancer is growing [1,2,3]. IMPT has the potential to lower the occurrence of radiation-treatment-related toxicities such as dysphagia and xerostomia [4]. In the current clinical practice, IMPT HN plans are generated using a constant relative biological effectiveness (cRBE) of 1.1 [5]. However, studies [5,6] indicate that the RBE can exhibit variability based on factors such as dose, treatment technique, cell type, radiosensitivity of the tissues, α/β ratio, oxygenation, clinical or biological endpoint, and linear energy transfer (LET). Numerous phenomenological models incorporating variable relative biological effectiveness (vRBE) have been developed [7,8].

Studies have shown that the RBE can be correlated to the dose-averaged LET (LET_d_) for therapeutic proton energies [9,10]. The highest LET_d_ in the proton plan occurs at the very distal part of the spread-out Bragg peak (SOBP) [11]. More recently, Underwood et al. [12] reviewed clinical studies on vRBE in proton therapy. Out of twenty-two studies reviewed by Underwood et al. [12], six studies concluded that they had found clinical evidence for vRBE, whereas twelve studies showed inconclusive results, and four studies did not find clinical evidence for vRBE. Several investigators [13,14,15,16,17,18,19] have delved into the dosimetric effects of vRBE models for various disease sites in IMPT. The vRBE along the path of protons presents an additional factor contributing to the uncertainty in IMPT planning. The increasing LET_d_ can lead to higher RBE-weighted dose (RWD) distributions [11,20]. Moreover, uncertainties in proton range could further influence the LET_d_ distributions [21,22,23], thereby impacting the RWD to the OARs located in close proximity to the clinical target volume (CTV).

While previous investigations [13,14,15,16,17,18,19] have explored the dosimetric impact of vRBE models for various cancers, the impact of combining proton range uncertainties and vRBE models in bilateral HN IMPT planning remains largely unexplored. Investigating the combined effect of proton range uncertainties and vRBE models for bilateral HN cancer is critical for several reasons. Firstly, bilateral HN cancers involve tumors situated near OARs, making accurate dose delivery essential to minimize toxicity and maintain treatment efficacy. Secondly, the use of IMPT allows for highly conformal dose distributions, but uncertainties in proton range and vRBE introduce complexities that can affect treatment outcomes. Thirdly, HN cancer patients often experience radiotherapy-induced side effects, such as xerostomia and dysphagia [24,25,26], highlighting the importance of optimizing treatment plans to minimize the dose to the surrounding critical structures. Therefore, the primary goal of the current study was to explore the influence of proton range uncertainties on the RWD to the OARs in the context of bilateral HN cancer treatment plans generated by employing the IMPT technique.

## 2. Materials and Methods

### 2.1. Contouring and Treatment Planning

In this Institutional Review Board (IRB)-approved retrospective treatment planning study, we included anonymized computed tomography (CT) datasets of ten bilateral HN cancer patients who had undergone photon therapy. The CTV was categorized into CTV_High and CTV_Low. The OARs were contoured using Limbus AI (v1.7; Limbus AI Inc., Regina, SK, Canada) software and later reviewed by the treatment planners.

Treatment plans were created within RayStation (v11B; RaySearch Laboratories, Stockholm, Sweden). We utilized the beam model that is based on the IBA’s ProteusOne PBS proton therapy system. Specifically, for each patient, a nominal IMPT treatment plan was generated using the Monte Carlo algorithm for both robust plan optimization (setup error: ±3 mm; range error: ±3%; 10,000 ions/spot) and final dose calculations (0.5% statistical uncertainty; 3 mm voxel size). Each plan included three beams with a range shifter of 4 cm water equivalent thickness (WET) to deliver doses to the CTV_High and CTV_Low for doses of 70 Gy(RBE) and 54 Gy(RBE), respectively, in 35 fractions through a simultaneous integrated boost (SIB) technique. All nominal plans used cRBE of 1.1. Plan normalization was performed to ensure that 99% of CTV_High received 69.3 Gy(RBE). Nominal plans were evaluated based on the institutional criteria for bilateral HN cancer.

### 2.2. Range Uncertainties

Each nominal plan calculated with a cRBE of 1.1 was subjected to the range uncertainties of ±3%. The range uncertainty in RayStation was modeled by scaling the mass density from the CT of the patient and was universal for all beams. The range uncertainties of −3% and +3% mean that the mass density was scaled by −3% and +3%, respectively.

### 2.3. LET_d_ and RWD Calculations

The LET_d_ distributions were calculated (3 mm voxel size) for the nominal and perturbed plans (±3% range uncertainty). In RayStation, LET_d_ is defined as the voxelwise unrestricted, dose-averaged LET_d_ [21]. The RWD was then calculated for the CTVs and OARs (see Table 1). For RWD calculations, we used the phenomenological vRBE model developed by McNamara [9].

(1) RBEmaxLETd, αβx=p0+p1αβxLETd,

(2) RBEminLETd, αβx=p2+p3  αβx LETd

where p_0_, p_1_, and p_2_ are the fit parameters (p_0_ = 0.99064, p_1_ = 1.1012, and p_2_ = −0.0039) in the McNamara model. The McNamara model is based on the linear quadratic model and incorporates the LET_d_ distributions within the specified volume. The McNamara model was chosen for this study due to its extensive application in exploring proton RBE effects in the recent literature [19,21,27,28,29]. Comprehensive details on the McNamara model are available in the referenced publications [7,9]. The α/β ratios can vary for different tumors and critical structures; however, for this study, we utilized the α/β values consistent with those used in RWD-related publications [19,21,27,28]. Specifically, an α/β ratio of 10 was applied to the CTVs, while an α/β ratio of 2 was used for all OARs. All RWD calculations were performed within the scripting environment of RayStation.

### 2.4. Dosimetric Analysis

For each patient, the differences (Δ) between the RWD distributions (*D_vRBE_*) and nominal dose distribution (i.e., *D_cRBE_*) were compared. Specifically, the mean dose and the maximum dose to the OARs and CTVs were evaluated per institutional protocol.

(3) $$\Delta \left(x\right)=D_{vRBE}\left(x\right)-D_{cRBE}\left(x\right),$$

(4) $$\Delta_{avg}\left(x\right)=\frac{1}{10}\sum_{i=1}^{10}D_{vRBE}\left(x\right)-D_{cRBE}\left(x\right)$$

where x = mean dose or maximum dose result.

## 3. Results

**Figure 1:**
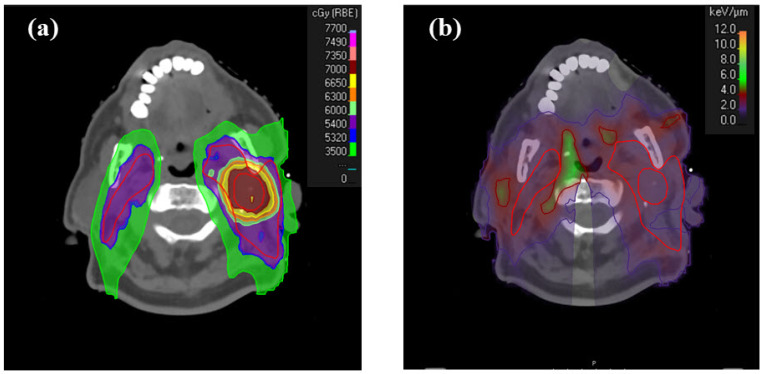
(**a**) Dose distributions (cRBE = 1.1) in a nominal plan (no range uncertainty) of an example HNC patient. (**b**) LET_d_ distributions in a nominal plan of the same patient shown in (**a**).

Table 1 shows the dosimetric differences between the RWD distributions and nominal dose distributions. Figure 1 illustrates the dose distributions (cRBE = 1.1) in a nominal plan (no range uncertainty) of an example HN patient as well as the LET_d_ distributions in a nominal plan of the same patient. The differences are averaged over ten patients, as shown in Equation (4).

### 3.1. Mean Dose (D_mean_) Evaluation for OARs

The vRBE model consistently yielded a higher mean dose to the OARs compared to the cRBE model. Within the vRBE model, the D_mean_ values exhibited variations among the three RWD distributions. Specifically, in a nominal scenario without the range uncertainty, the D_mean_ was higher than that in a +3% range uncertainty scenario but lower than that in a −3% range uncertainty scenario. The Δ_avg_ for the esophagus and oral cavity ranged from 2.76 to 3.40 Gy(RBE) and from 3.33 to 4.00 Gy(RBE), respectively. Analysis of the left and right parotids revealed Δ_avg_ values ranging from 3.72 to 5.02 Gy(RBE). Additionally, for the larynx and thyroid, the Δ_avg_ ranged from 5.18 to 6.91 Gy(RBE) and from 6.54 to 6.90 Gy(RBE), respectively.

### 3.2. Maximum Dose (D_max_) Evaluation for OARs

The evaluation of D_max_ exhibited a similar trend to that of D_mean_, with the vRBE model predicting higher values compared to the cRBE model. Additionally, a consistent pattern was observed where the vRBE model’s results for a nominal scenario were higher compared to a +3% range uncertainty scenario but lower compared to a −3% range uncertainty scenario. For the brachial plexus, the Δ_avg_ was up to 10.75 Gy(RBE). In the case of the brain and brainstem, the Δ_avg_ ranged from 4.93 to 6.59 Gy(RBE) and from 2.78 to 3.86 Gy(RBE), respectively. Regarding the spinal cord, the Δ_avg_ ranged from 2.13 to 5.19 Gy(RBE). However, for the mandible, the Δ_avg_ remained consistent at 7.10–7.20 Gy(RBE) for both nominal and range uncertainty scenarios.

### 3.3. D_mean_ and D_max_ Evaluation for Target Volumes

**Figure 2:**
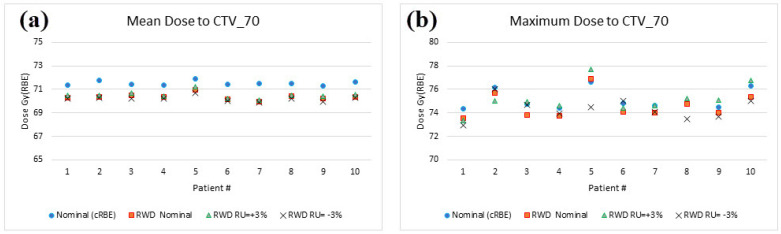
(**a**) Mean dose to the CTV_70 and (**b**) maximum dose to the CTV_70 in nominal (cRBE), RWD of nominal, RWD of +3% range uncertainty, and RWD of −3% range uncertainty plans in ten patients.

Figure 2 illustrates the D_mean_ and D_max_ to CTV_70 in ten patients for cRBE and vRBE models. For D_mean_, the vRBE model always produced lower values compared to the cRBE model. On average, the D_mean_ from RWD distributions (vRBE) was lower by 1.6 ± 0.3% for a nominal (i.e., unperturbed) scenario, 1.4 ± 0.4% for a range uncertainty of +3% scenario, and 1.8 ± 0.2% for a range uncertainty of −3% scenario. In evaluating D_max_ results in all ten patients, there was no clear trend of the vRBE model producing either higher or lower than the cRBE model for different scenarios. On average, when compared to the cRBE model, the D_max_ from the RWD distribution (vRBE) was lower by 0.7 ± 0.5% for a nominal (i.e., unperturbed) scenario, 0.1 ± 0.9% for a range uncertainty of +3% scenario, and 1.1 ± 1.0% for a range uncertainty of −3% scenario.

## 4. Discussion

The current clinical practice involves generating IMPT plans using a cRBE of 1.1. However, it is known that the RBE can vary based on several factors, leading to the development of models incorporating vRBE [7]. Our study contributes to the evolving understanding of vRBE in IMPT for HN cancer. Specifically, we explored the influence of proton range uncertainties on the RWD to the OARs and target volumes in bilateral HN cancer treatment plans generated using the IMPT technique.

The results from our study revealed notable variations in D_mean_ values for the majority of OARs between the cRBE and vRBE models. The vRBE model consistently yielded higher D_mean_ values, with variations observed among different range uncertainty scenarios. The constrictor muscles, oral cavity, parotids, larynx, thyroid, and esophagus showed large differences in D_mean_ values, indicating the impact of proton range uncertainties on RWD distributions. Similarly, the evaluation of D_max_ exhibited a consistent trend, with the vRBE model predicting higher values compared to the cRBE model. The brachial plexus, brain, brainstem, spinal cord, and mandible showed varying degrees of differences in D_max_ values under different scenarios, highlighting the sensitivity of these structures to proton range uncertainties. Incorporating insights from other studies, such as the work by Garbacz et al. [27] and Hahn et al. [21], reinforces our findings. Hahn et al.’s study [21] observed the LET_d_ hotspots in OARs, particularly in brain tumor patients. Garbacz et al. [27] highlighted that the challenges in treating tumors that are close to critical structures necessitate careful consideration of vRBE to avoid increased doses and extended beam ranges.

In assessing the CTVs, Rana et al. [22] and Hahn et al. [21] emphasized the importance of considering range uncertainties and highlighted the robustness of LET_d_ distributions in the target volumes. Our study demonstrated that the vRBE model consistently produced lower D_mean_ values to the CTVs compared to the cRBE model. However, the trend in D_max_ values for the CTV was less clear, with no consistent pattern observed across range uncertainty scenarios.

A comprehensive review by Underwood et al. [12] explored the existing literature on vRBE in proton therapy, shedding light on the current state of knowledge in this field. The review considered twenty-two studies, revealing a variety of conclusions. Only six studies provided clinical evidence supporting the presence of vRBE, while twelve studies presented inconclusive results, and four studies did not find supporting evidence. Our study was performed based on the McNamara vRBE model for RWD calculations. Rorvik et al.’s study [12] highlighted considerable variations in estimations of RBE and RWD from different models. This emphasizes the importance of acknowledging these variations in the treatment planning study. In our study, the use of the SIB technique for treating HNC results in varying fractional doses—both high and low—was considered. Previous reports [30] indicate that a lower fractional dose can potentially increase the RBE. Future studies should explore how the combination of range uncertainty and different fractionation schemes can impact the RWD dose to the OARs. Additionally, we used the alpha/beta ratio of 2 for all the OARs. Resch et al. [31] provided insight into the uncertainty of alpha/beta ratios in nasopharyngeal and prostate cancer cases and their OAR overdosage. The findings from the above-mentioned studies [7,12,31] align with our emphasis on the need for standardized approaches in interpreting results from different RBE models. Moreover, the current ambiguity on clinical evidence of vRBE underscores the challenges in establishing a consistent understanding of the impact of variable RBE in proton therapy.

## 5. Conclusions

The consistent trend of higher mean and maximum doses to the OARs with the McNamara vRBE model compared to cRBE model highlighted the need for consideration of proton range uncertainties while evaluating OAR doses for bilateral HN cancer treatment plans generated by employing the IMPT technique.

## Figures and Tables

**Table 1:** Dosimetric differences in D_mean_ and D_max_ between the RWD and nominal dose distributions.

	Δ_avg_						
	D_mean_ [Gy(RBE)]				D_max_ [Gy(RBE)]		
OARs	Nominal	RU = +3%	RU = −3%		Nominal	RU = +3%	RU = −3%
Brachail Plexus_L	-	-	-		10.44	10.18	10.75
Brachical Plexus_R	-	-	-		9.86	9.65	10.36
Brain	-	-	-		5.80	4.93	6.59
Brainstem	-	-	-		3.14	2.78	3.86
Constrictor Muscles	6.75	5.59	7.85		-	-	-
Esophagus	2.76	2.09	3.40		-	-	-
Larynx	6.06	5.18	6.91		-	-	-
Mandible	-	-	-		7.17	7.13	7.10
Oral Cavity	3.33	2.60	4.00		-	-	-
Parotid_L	4.59	4.11	5.02		-	-	-
Parotid_R	4.02	3.72	4.27		-	-	-
Spinal Cord	-	-	-		3.53	2.13	5.19
Thyroid	6.72	6.54	6.90		-	-	-

Note: Δ_avg_ is calculated using Equation (4); RWD = RBE-weighted dose; and RU = range uncertainty.

## Data Availability

Requests to access the datasets should be directed to the corresponding author.
